# Factors associated with oesophageal cancer in Soweto, South Africa.

**DOI:** 10.1038/bjc.1988.286

**Published:** 1988-11

**Authors:** I. Segal, S. G. Reinach, M. de Beer

**Affiliations:** Gastroenterology Unit, Baragwanath Hospital, South Africa.

## Abstract

Cancer of the oesophagus was a rare disease in the South African black population until the last few decades. Increases in incidence have occurred and at present it is the commonest cancer in black men in many parts of South Africa. A case-control study of 200 oesophageal cancer patients and 391 hospital controls has been carried out in order to determine the risk factors in the urban black population of Soweto. The results indicate that the cancer patients were long-term urban residents from a very low socio-economic group. The association between smoking pipe tobacco and oesophageal cancer previously noted in South Africa is confirmed. In addition, consumption of traditional beer was found to be a major risk factor.


*Br. J. Cancer* (1988), **58**, 681–686. © The Macmillan Press Ltd., 1988

### Factors associated with oesophageal cancer in Soweto, South Africa

I. Segal[1], S.G. Reinach[2] & M. de Beer[2]

[1]*Gastroenterology Unit, Baragwanath Hospital and University of Witwatersrand, Johannesburg, South Africa and* [2]*Institute for Biostatistics of The South African Medical Research Council, South Africa.*

**Summary** Cancer of the oesophagus was a rare disease in the South African black population until the last few decades. Increases in incidence have occurred and at present it is the commonest cancer in black men in many parts of South Africa. A case-control study of 200 oesophageal cancer patients and 391 hospital controls has been carried out in order to determine the risk factors in the urban black population of Soweto. The results indicate that the cancer patients were long-term urban residents from a very low socio-economic group. The association between smoking pipe tobacco and oesophageal cancer previously noted in South Africa is confirmed. In addition, consumption of traditional beer was found to be a major risk factor.

Cancer of the oesophagus was an uncommon disease in the South African black population during the 1920s and 1930s. Since then an alarming increase in incidence has occurred (Higginson & Oettlé, 1960; Oettlé, 1963; Rose, 1973). In some parts of the country it is now the most common cancer in black men (Rose, 1973). Figures from hospitals in Johannesburg which have served the black population show an increase from 2% of all tumours in men in the 1930s, to 11% in the early 1950s and 28% in the early 1960s (Cook, 1971). In Soweto, which is adjacent to Johannesburg and where most of the black population are resident, standard annual incidence rates during 1980–1982 for males and females were 26 and 6 per 100,000 (standardised to world population) respectively (Walker *et al.*, 1984). This is on a par with figures previously published for blacks in Cape Province and slightly below those for Natal (Doll *et al.*, 1970). The highest incidence rates that have been reported from Southern Africa are still those for the south of the Transkei, 63 per 100,000 for males in 1981 and 65 for females (Van Rensburg *et al.*, 1983).

Studies of western populations have indicated a multiplicative effect of alcohol and tobacco in the development of cancer of the oesophagus with risk increasing more sharply with rising alcohol intake than with rising tobacco consumption (Tuyns *et al.*, 1977; Day, 1984). In France, Barrelier (1974) has suggested a geographical association with home-distilled drinks. Previous case-control studies among South African black populations have shown an elevated risk for the smoking of pipe tobacco and more recently for the smoking of cigarettes but no evidence for an independent risk associated with the consumption of alcohol (Bradshaw & Schonland, 1969; 1974; Van Rensburg *et al.*, 1985). However a population survey conducted in areas of high and low incidence in the Transkei suggests that a combined effect of smoking pipe tobacco and of drinking may be of importance there (McGlashan *et al.*, 1982). Cook (1971) collated information on the occurrence of cancer of the oesophagus throughout Africa and found evidence for a geographical and temporal association with the consumption of beer made from maize. In South Africa there had been a change from using mostly sorghum for beer-making to using mostly maize with sorghum retained only as the fermenting agent. Bradshaw *et al.* (1983) noted that sorghum continued to be used as the malt for homebrewed beer longer in the low incidence areas of the north of the Transkei than in the high incidence areas in the south.

Enigmas in terms of risk factors arise when one considers the high incidence areas of northern Iran, China and Soviet Central Asia. Alcohol and tobacco are not important aetiological factors in these regions (Joint Iran–IRAC study group, 1977). Risk was found to be highest in the poor sections of the community (Cook-Mozaffari *et al.*, 1979) and dietary deficiencies have been observed in all these areas (Hormozdiari *et al.*, 1975; Kolichava, 1980 and Thurnham *et al.*, 1985). Van Rensburg (1981) has investigated the type of dietary deficiency that is common in areas of high incidence and has suggested that low intakes of riboflavin, nicotinic acid, magnesium and zinc are the important factors. A study conducted in the United States among patients who had a high alcohol intake demonstrated poor nutritional intake (Pottern *et al.*, 1981) and it seems likely that inadequate nutrition is an underlying factor in all populations with a high risk of oesophageal cancer (Day & Munoz, 1982).

Against this background a case-control study was carried out among residents of Soweto to determine risk factors present in an urban black population whose life style has changed considerably since the first case-control study was conducted in the mid-sixties (Bradshaw and Schonland, 1974) and quite dramatically compared with their rural counterparts.

### *Background*

The population of Soweto is one and a half million or more. The population is relatively balanced in terms of males and females and has a young age structure indicative of the current high population growth rate. The average family size is 5.3 persons. The number of occupants per house is estimated to be about 10. At least 18.7% of households live below the subsistence level (Morris & Celliers, 1980; Morris, 1981).

Soweto is inhabited by different ethnic communities, the main groups being Zulu (33%), Tswana (16.3%), Sesuthu (13.7%) and Xhosa (10.3%). Bradshaw and Schonland (1974) found an elevated risk in the Xhosa and Tswana groups. An increasing proportion of the population will have been born and lived all their lives in Soweto.

Baragwanath Hospital and various polyclinics provide the essential health services for Soweto. Baragwanath Hospital, with 2,740 beds is the largest hospital on the African continent. The total number of admissions for the 1 year period 1 April 1982–31 March 1983 was 122,610. The number of outpatients (including polyclinic patients) during this period was 1,637,956.

### Methods

### *Patients*

Two hundred consecutive patients with oesophageal cancer who were resident in Soweto were interviewed during 1984

Correspondence: I. Segal.
Received 29 December 1987; and in revised form, 7 April 1988.

and 1985. Previous experience with cancer survival studies suggests that at least 20% of patients may give incorrect addresses or cannot be traced once they leave hospital (Walker *et al.*, 1984). All patients had histologically proven squamous cancer.

*Controls*

Three hundred and ninety-one hospital controls were interviewed. The choice of controls was directed towards diseases in which alcohol and smoking were thought to play no part in aetiology. Thus patients with the following diagnoses were chosen – inguinal hernias, appendicitis, benign prostatic hypertrophy, cataracts (in non-diabetics) and haemorrhoids. Patients with cancer at other sites excluding oesophagus were not included as controls. The reason for this was to obviate any environmental factors which could be implicated in these cancers and cancer of the oesophagus.

Each patient was matched to approximately two controls of the same sex but not of the same age. Patients and controls were selected by one of the authors (IS). Interviews were conducted in hospital by a black nursing sister, trained in interviewing techniques, under the supervision of IS. The questionnaire was written in English.

*Questionnaire*

The questionnaire was designed to focus retrospectively on (1) alcohol intake, (2) smoking, (3) indices of socio-economic status as a surrogate for probable poor nutrition and (4) length of urbanisation.

(1) *Alcohol intake* Traditionally, beer (either brewed at home, or brewed commercially using local recipes) was, until fairly recently, the main type of alcohol imbibed by South African blacks. It is low on alcohol content ($\pm 3\%$) and made from malted sorghum and a starchy adjunct – sorghum grain or maize (Novellie, 1968). There has, however, been a marked increase in the consumption of commercially available spirits among South African blacks (Segal *et al.*, 1984*a*,*b*). This has happened since 1961 when the legislation was repealed that made it illegal for blacks to purchase western spirits. It is emphasised however that home-distilling of liquor still takes place in Soweto. The extent of this practice is not known. Subjects were questioned as to the type and quantity of alcohol consumption and about the fermenting agent and flour used for the manufacture of home-brewed beer. No distinction was made in the questioning between home-brewed beer and commercial African beer. In the text both types will be referred to as traditional beer.

(2) *Smoking* Initially, questions were included about the number of cigarettes smoked and about pipe smoking. Subsequently the survey was extended to include questions about the use of hand-rolled cigarettes in which pipe tobacco is used wrapped in newspaper, brown paper or telephone directory paper. Such cigarettes were often preferred because of their flavour and their slow burning quality. They were also cheaper than commercial cigarettes. A further 96 oesophageal cancer and 184 controls were interviewed about the type of cigarettes that they smoked using the same criteria for selection as those listed above.

(3) *Indices of socio-economic status* Patients and controls were questioned as to education, occupations, salaries, ownership of cars, bicycles, refrigerators and television sets.

(4) *Urbanisation* Questions were included on birthplace and number of years of residence in Soweto.

*Statistical procedures*

For categorical data the Chi-squared test was used to compare the cases and controls and with continuous data the *t*-test was used (SAS, 1986). The significant results are set out in Table I*a*,*b*.

Stepwise logistic regression was used to determine the major risk factors for oesophageal cancer (SUGI, 1986) and to calculate the estimates of relative risk (RR). RRs for each of the variables included in the analysis (with allowance made for each of the others) are given in Table II*a*,*b* separately for men and women. No allowance was made for matching. The order in which the variables were fed into the logistic regression analysis were directly dependent on the quoted probability values and these probabilities also indicates the significance of the predictors (Tables II*a*,*b* & III). In Table IV the combined RRs were calculated using methods described by Breslow and Day (Breslow & Day, 1980).

### Results

*Sex*

Seventy-five percent of cancer patients were men and 25% were women. Seventy-nine percent of controls were men and 21% were women (Table I*a*).

*Age*

The mean age of the cancer patients was 55 years and the controls 59 years (cancer male patients 56.7 years, controls 60 years; cancer female patients 50.7 years, controls 57.6 years). Comparisons, have, therefore, been standardised for age and age has been included as a factor in the logistic regression analyses (Table I*a*,*b*).

*Birthplace and residence in Soweto*

Twenty-three percent of patients and 18% of controls were born in the city (Table I*a*). The mean period of residence in Soweto for the patients and controls was 36.7 and 36.1 years respectively.

*Ethnic group*

Cancer affected all the different ethnic groups living in Soweto with no difference between the groups (Table I*a*).

*Educational status*

There was no significant difference in the educational standards between the patients and controls. Education standards were low in both groups. Only 22% of controls and 19% of patients had received some form of high school education (Table I*a*).

*Salaries*

The patients earned significantly lower salaries compared with the controls (approximately 31 Rand and 49 Rand weekly respectively; $P<0.0001$) (Table I*a*,*b*). Salary level emerges as one of the major associated factors for development of cancer of the oesophagus with an increase in risk with decreasing salary for both men and women (Table II*a*,*b*).

*Ownership of material possessions*

Significantly more controls owned motor cars and television sets than patients (motor cars; controls 20%, patients 9%; $P<0.001$). Television sets: controls 40%, patients 25%; $P<0.001$). Forty-four percent of the controls and 36% of the cases owned refrigerators, but this difference was not statistically significant (Table I*a*).

*Marital status*

There were significant differences in the number of married and single patients in the two groups. More controls were

**Table I** (A) Characteristics of cases and controls

| | *Cases* | *Controls* | *Crude relative risk* | *95% Confidence interval* | *P value*[a] |
|---|---|---|---|---|---|
| Sex: | | | | | |
| Male | 150 (75%) | 309 (79%) | | | 0.266 |
| Female | 50 (25%) | 82 (21%) | | | |
| Age: | | | | | |
| mean ± s.e.[b] | 55±0.830 | 59±0.637 | | | |
| range | (27–90) | (25–88) | | | 0.0001 |
| Birthplace: | | | | | |
| Urban | 46 (23%) | 70 (18%) | 1.34 | (0.9–2.0) | 0.170 |
| Ethnic group: | | | | | |
| Xhosa | 37 (19%) | 52 (14%) | | | |
| Zulu | 41 (21%) | 78 (21%) | | | |
| Sotho | 32 (17%) | 55 (15%) | | | |
| Tswana | 46 (24%) | 68 (18%) | | | |
| Education: High school | 37 (19%) | 86 (22%) | 0.81 | (0.5–1.2) | 0.322 |
| Salary per week: | | | | | |
| mean ± s.e.[b] | R31.00±2.128 | R49.00±2.205 | | | 0.0001 |
| range | (0.00–R150.00) | (0.00–R162.00) | | | |
| Material possessions: | | | | | |
| Motor car | 17 (9%) | 79 (20%) | 0.37 | (0.2–0.6) | <0.001 |
| Television | 50 (25%) | 156 (40%) | 0.50 | (0.3–0.7) | <0.001 |
| Refrigerator | 72 (36%) | 161 (44%) | 0.71 | (0.5–1.0) | 0.059 |
| Marital status: | | | | | |
| Married | 104 (52%) | 266 (68%) | 0.51 | (0.4–0.7) | <0.001 |
| Single | 34 (17%) | 22 (6%) | 3.44 | (1.9–6.1) | <0.001 |
| Divorced | 20 (10%) | 16 (4%) | 2.60 | (1.3–5.1) | 0.004 |
| Cigarette smoking | 149 (75%) | 180 (46%) | 3.42 | (2.4–5.0) | <0.001 |
| Pipe smoking | 50 (25%) | 47 (12%) | 2.43 | (1.6–3.8) | <0.001 |
| Pipe smoking[c] | 21 (22%) | 12 (7%) | 4.01 | (1.9–8.6) | <0.001 |
| Only commercial cigarettes[c] | 30 (31%) | 55 (30%) | 1.07 | (0.6–1.8) | 0.814 |
| Only hand-rolled cigarettes[c] | 10 (10%) | 7 (4%) | 2.84 | (1.1–8.0) | 0.028 |
| Commercial and hand-rolled cigarettes[c] | 40 (42%) | 18 (10%) | 6.6 | (3.5–12.4) | <0.001 |
| Traditional beer consumption | 159 (80%) | 169 (43%) | 5.09 | (3.4–7.6) | <0.001 |
| Quantity of traditional beer: l/week | | | | | |
| mean ± s.e.[b] | 12.0±0.580 | 4.4±0.344 | | | |
| range | (0.0–45.0) | (0.0–31.5) | | | |
| Duration of drinking: | | | | | |
| mean ± s.e.[b] | 26.4±1.444 | 17.2±1.066 | | | |
| range | (0.0–64.0) | (0.00–58.00) | | | 0.0001 |
| Flour for beer: | | | | | |
| Maize flour | 160 (80%) | 165 (42%) | 5.46 | (3.7–8.1) | <0.001 |
| Fermenting agent in traditional beer: | | | | | |
| Bakers yeast | 72 (36%) | 38 (10%) | 5.18 | (3.3–8.1) | <0.001 |
| Western spirit consumption | 121 (61%) | 111 (28%) | 3.86 | (2.7–5.5) | <0.001 |
| Quantity of commercial spirits: l/week | | | | | |
| mean ± s.e.[b] | 0.55±0.046 | 0.22±0.040 | | | |
| range | (0–13.3) | (0–4) | | | |

[a]*P* value from Chi-square test or Students *t* test; [b]s.e. = standard of mean; [c]From the second study with 94 cases and 184 controls.

**Table I** (B) Characteristics of cases and controls for males and females separately

| | *Males* | | | *Females* | | |
|---|---|---|---|---|---|---|
| | *Cases* | *P value*[a] | *Controls* | *Cases* | *P value*[a] | *Controls* |
| Age: | | | | | | |
| mean ± s.e.[b] | 56.7±0.925 | | 60.0±0.690 | 50.74±1.682 | | 57.55±1.563 |
| range | (28–90) | 0.0058 | (29–88) | (27.00–70.00) | 0.0052 | (25.00–84.00) |
| Salary per week: | | | | | | |
| mean ± s.e.[b] | R37.03±2.529 | | R52.50±2.359 | R10.58±2.128 | | R32.13±5.185 |
| range | (R0.00–R150.00) | 0.0001 | (R0.00–R162.00) | (R0.00–R80.00) | 0.0003 | (R0.00–R150.00) |
| Alcohol consumption: ($l\,week^{-1}$) | | | | | | |
| Traditional beer: | | | | | | |
| mean ± s.e.[b] | 13.39±0.635 | | 5.04±0.406 | 7.63±1.128 | | 2.155±0.518 |
| range | (0.00–45.00) | 0.0001 | (0.00–31.50) | (0.00–31.00) | 0.0001 | (9.99–22.50) |
| Western spirits: ($l\,week^{-1}$) | | | | | | |
| mean ± s.e.[b] | 0.67±0.055 | | 0.26±0.049 | 0.20±0.386 | | 0.04±0.017 |
| range | (0.00–4.00) | 0.001 | (0.00–13.25) | (9.00–1.75) | 0.0100 | (0.00–0.75) |
| Total alcohol consumption: ($g\,day^{-1}$) | | | | | | |
| mean ± s.e.[b] | 86.57±4.196 | | 31.36±2.467 | 40.06±6.445 | 0.0001 | 10.71±2.542 |
| range | (0.00–250.35) | 0.0001 | (0.00–299.05) | (0.00–210.31) | | (0.00–96.53) |
| Cigarette smoking: (number $day^{-1}$) | | | | | | |
| mean ± s.e.[b] | 8.57±0.681 | | 4.73±0.352 | 3.90±0.759 | | 1.06±0.342 |
| range | (0.00–50.00) | 0.0001 | (0.00–38.00) | (0.00–20.00) | 0.0011 | (0.00–15.00) |

[a]*P* value from Student's *t* test; [b]s.e. = standard error of mean.

**Table II** (A) Relative risk for various factors associated with oesophageal cancer in men

| *Variable* | *Groups compared* | *Relative risk* | *95% Confidence interval* | *P value*[a] |
|---|---|---|---|---|
| Quantity traditional beer ($l\,week^{-1}$) | 15 l *vs* nil | 3.90 | (2.2–6.9) | 0.0001 |
| | 10 l *vs* nil | 2.48 | (1.7–3.6) | |
| | 6 l *vs* nil | 1.57 | (1.3–1.9) | |
| | 2.5 l *vs* nil | 1.25 | (1.1–1.4) | |
| Salary (Rand) | Nil *vs* R100 | 5.70 | (2.2–14.5) | 0.0003 |
| | Nil *vs* R60 | 2.84 | (1.6–5.0) | |
| | Nil *vs* R40 | 2.01 | (1.4–3.0) | |
| | Nil *vs* R12 | 1.23 | (1.1–1.4) | |
| Quantity commercial spirits ($l\,week^{-1}$) | 3 l *vs* nil | 7.20 | (1.6–33.2) | 0.0114 |
| | 2 l *vs* nil | 3.73 | (1.3–10.3) | |
| | 1.5 l *vs* nil | 2.68 | (1.2–3.2) | |
| | 1 l *vs* nil | 1.93 | (1.2–3.2) | |
| Cigarette smoking (number $day^{-1}$) | 20 *vs* nil | 2.73 | (1.3–6.0) | 0.0117 |
| | 15 *vs* nil | 2.13 | (1.2–3.8) | |
| | 10 *vs* nil | 1.65 | (1.1–2.4) | |
| | 5 *vs* nil | 1.29 | (1.1–1.6) | |
| Bakers yeast | Yes/No | 2.17 | (1.1–4.2) | 0.0234 |

Relative risk calculated from a single analysis incorporating all the above factors and the age of the subjects.
[a]*P* values from logistic regression analysis.

**Table II** (B) Relative risk for various factors associated with oesophageal cancer in women

| *Variable* | *Groups compared* | *Relative risk* | *95% Confidence interval* | *P value*[a] |
|---|---|---|---|---|
| Quantity traditional beer ($l\,week^{-1}$) | 15 l *vs* nil | 25.70 | (2.8–238.4) | 0.0043 |
| | 10 l *vs* nil | 8.71 | (2.0–38.5) | |
| | 1 *vs* nil | 2.95 | (1.4–6.2) | |
| | 2.5 l *vs* nil | 1.72 | (1.2–2.5) | |
| Salary (Rand) | Nil *vs* R60 | 74.94 | (2.7–83.3) | 0.0020 |
| | Nil *vs* R49 | 6.07 | (1.9–19.1) | |
| | Nil *vs* R12 | 1.72 | (1.2–2.4) | |

Relative risk calculated from a single analysis incorporating all the above factors and the age of the subjects.
[a]*P* values from logistic regression analysis.

married (68% controls, 52% patients; $P<0.001$) and fewer were single (6% controls, 17% patients; $P<0.001$). Also a greater proportion of patients were divorced (10% patients, 4% controls; $P=0.004$) (Table I*a*).

*Smoking*

*Initial study* Seventy-five percent of patients smoked cigarettes compared to 46% controls ($P<0.001$). Twenty-five percent either smoked only pipes or smoked both a pipe and cigarettes compared to 12% controls ($P<0.001$) (Table I*a*). The patients smoked 7.4 cigarettes and controls 4 cigarettes daily ($P=0.0001$).

*Extended study* (An additional 96 patients and 184 controls questioned in order to determine use of hand-rolled cigarettes). Forty-two percent of the cases and 10% of the controls smoked commercial and hand-rolled cigarettes and of these 10% of patients and 4% controls smoked only hand-rolled cigarettes. Twenty-two percent of the cases and 7% of the controls were pipe smokers. The crude RR indicates that there is no risk involved in smoking only commercial cigarettes. (RR 1.07, Table I*a*). However in conjunction with other predictors it becomes an important risk factor. Risk increased with increasing consumption for the smokers of both hand-rolled and commercial cigarettes but the greatest increase was associated with the relatively modest use of hand-rolled cigarettes (RR 13.5 associated with an average consumption of 6 per day) (Table III). However, these RRs cannot be compared directly with that shown in Table I*a*,*b* because no allowance can be made for other risk factors.

*Traditional beer consumption*

Eighty percent of patients drank traditional beer as compared with 43% of controls ($P<0.001$). The mean duration of drinking by patients was 26.4 years, controls 17.2 years ($P=0.0001$). The mean quantity of traditional beer consumed was 12 l weekly, controls 4.4 l weekly ($P=0.0001$). Maize meal was used as flour for beer by 80% of patients and 42% of controls ($P<0.001$) (Table I*a*). When levels of consumption are considered, there is a clear increase in risk with increasing quantity consumed for both men and women after allowance for other factors (Table II*a*,*b*).

*Western spirit consumption*

Sixty-one percent of patients drank western spirits, compared with 28% of controls ($P<0.001$). The mean quantity of spirits consumed was 0.55 l weekly (controls, 0.22 weekly) (Table I*a*). Risk increased with increasing consumption for men (only 30% of women patients consumed spirits) with a high RR (7.2) for those who consumed an average of 3 l per week (Table II*a*). It should be noted that the RR was obtained after controlling for other factors in Table II*a*, as well as for age.

*Joint effects of smoking and alcohol consumption*

Table IV shows the joint effect of the two factors which are multiplicative. The RR associated with heavy consumption of both is extremely high. Men who smoked more than 40 g of tobacco and drank more than 91 g of alcohol daily had a RR of 39. This compares with a RR of unity for men who smoked less than 19 g of tobacco and drank less than 30 g of alcohol per day.

### Discussion

The results of the current study indicate that males are

**Table III** Relative risk of smoking hand-rolled cigarettes, commercial cigarettes and pipes (based on 2nd study carried out on 96 cases and 184 controls)

| *Variable* | *Groups compared* | *Relative risk* | *95% Confidence interval* | *P value*[a] |
|---|---|---|---|---|
| Hand-rolled cigarettes (number day$^{-1}$) | 6 *vs* nil | 13.45 | (4.2–37.3) | |
| | 4 *vs* nil | 5.67 | (2.9–11.2) | <0.0001 |
| | 2 *vs* nil | 2.38 | (1.7–3.3) | |
| Commercial cigarettes (number day$^{-1}$) | 20 *vs* nil | 5.99 | (1.8–19.8) | |
| | 15 *vs* nil | 3.83 | (1.6–9.4) | 0.0033 |
| | 10 *vs* nil | 2.45 | (1.4–4.5) | |
| | 5 *vs* nil | 1.56 | (1.2–2.1) | |
| Pipe smoking | Yes/No | 4.88 | (2.2–10.9) | 0.0001 |

Relative risk calculated from a single analysis incorporating the above factors. [a]*P* values from logistic regression analysis.

**Table IV** Relative risk for the combined effects of smoking and alcohol consumption

| *Tobacco g day$^{-1}$ smoked* | *Alcohol g day$^{-1}$ ethanol)* | | | | *Tobacco adjusted for alcohol* |
|---|---|---|---|---|---|
| | *0–30* | *31–60* | *61–90* | *≥91* | |
| 0–19 | 1.00 | 4.24 | 4.00 | 9.54 | 1.00 |
| 20–39 | 1.00 | 7.14 | 55.00 | 45.00 | 2.97 (1.5–5.9) |
| ≥40 | 0.72 | 8.21 | 10.48 | 38.97 | 2.18 (1.3–3.7) |
| Alcohol adjusted for tobacco | 1.00 | 5.44 (2.8–10.5) | 10.48 (5.6–19.6) | 18.28 (10.1–33.2) | |

In brackets the 95% confidence interval for the Mantel–Haenszel summary relative risk.

affected much more commonly than females. This is in accordance with previous studies from urban areas in South Africa (Hunt, 1978). The mean age of the patients, 55 years, is similar to that of a series from Johannesburg in 1962–1964 (Hunt, 1978). The patients were an urbanised group – the mean period of residence was 37 years and 23% were born in Soweto. All ethnic groups were equally affected whereas in the earlier study (Schonland & Bradshaw, 1974) the Xhosa and Tswana groups had a slightly elevated risk, this probably implies that differences of habit rooted in traditional regional customs are being lost with continued urbanisation.

Although there was no significant difference in education level between the two groups this must be seen in the context of Soweto where the general level of education is poor (Morris & Celliers, 1980; Morris, 1981). Salaries and acquisition of material possessions, however, showed that the controls were better off than the cancer patients. It is probable that the higher risk among single and divorced persons also reflects poor socio-economic status. The low socio-economic status of oesophageal cancer patients is in accord with studies from many other parts of the world (Day & Munoz, 1982). The significance of these results seems to lie in the poor diet that accompanies the poor living conditions.

In the present study the majority of patients (75%) were smokers, and smoked mainly cigarettes. The quantity smoked was, however, relatively low – less than 7 cigarettes daily compared with 30 cigarettes daily in South African whites (Coetzee AM, 1978). As in the previous study from Soweto, an elevated risk was associated with the smoking of pipe tobacco either in pipes or in cigarettes (Bradshaw & Schonland, 1974). However, only 25% of the patients were pipe smokers. This contrasts with previous studies where pipes were used more frequently by smokers, 62.8% of patients (males) in Soweto in the mid-60s smoked pipes (Bradshaw & Schonland, 1974) and 76.3% of the general male population in a high incidence area of the Transkei in the mid-1970s (McGlashan *et al.*, 1982). The change in smoking habits within Soweto probably reflects increasing urbanisation. A similar number (41.3% in the 1960s compared with 41.7% in the 1980s) smoked pipe tobacco in hand-rolled cigarettes. As in the mid-60s, no elevated risk was associated with the smoking of commercial cigarettes alone. It must be noted, however, that in the study carried out in the 1960s only men were included. In the current study men and women are reported.

The present study thus confirms the association between pipe tobacco and oesophageal cancer. It differs from other South African studies, however, in that smoking is not the only major risk factor associated with oesophageal cancer (Table II*a*,*b*) and so corroborates Day's statement that even though tobacco is clearly of importance the relative risk seen in case control studies was far from sufficient to explain the major differences in incidence in Southern Africa (Day, 1984).

The majority of patients (80%) drank traditional beer and this was found to be a major risk factor (Table II*a*,*b*). The drinking and the quantity of alcohol consumed were both significantly greater in the cancer group than in controls. This applied to both traditional beer and western liquor. It is of interest that as in studies from other parts of the world the increase in risk with alcohol consumption seems to be more than linear whereas that with cigarette smoking is less than linear (Day & Munoz, 1982). Furthermore the joint effect of smoking and drinking are multiplicative – there is a marked increase in risk with increasing consumption of tobacco and alcohol (Table IV). It has been suggested that alcohol might act as a solvent facilitating the passage of carcinogens to the lower layers of the oesophagus (Doll, 1971).

The risk associated with the consumption of traditional beer may not rest solely in the quantity of alcohol consumed. The increase in risk associated with the use of maize meal as the major ingredient of beer accords with the finding by Cook (1971) of an association in Africa with the use of maize for beer making. The traditional alcoholic drink in South African blacks is a beer low on alcohol content (±3%) made from malted sorghum and a starchy adjunct – sorghum grain or maize (corn) (Novellie, 1968). In fact maize has been an ingredient of beer even before the turn of the century (Novellie, 1986) but the percentage of maize used in beer has increased considerably in recent times. A typical recipe given by Oxford (1926) contains maize meal – 27.8% sorghum meal – 37.6% and sorghum malt – 34.6%. In 1964 however 57% of the content of traditional beer was derived from maize (Cook, 1971). The use of maize instead of sorghum grain has resulted in a decrease in the thiamine, niacin and riboflavin content of traditional brews (Zammit, 1980). This would have dramatic effects on the vitamin B status of people such as the oesophageal cancer patients in the present study who consume large quantities of beer and whose low socio-economic status will result in a generally poor diet that is also largely composed of maize (Van Rensburg, 1981). It is of interest that a recent study from Natal found a high RR (5.7) for those who bought maize daily compared with those who bought it less than once a week (Van Rensburg *et al.*, 1985). There is no evidence to suggest that carcinogens in home-brewed beer or in home-distilled spirits from other parts of the world are of any importance in the development of cancer of the oesophagus (IARC Annual Reports, 1975 and 1976).

Yeast, used in the fermentation of home-brewed beer, emerged as a risk factor for oesophageal cancer (Table II*a*). The implications of this finding are unclear. It is not suggested that yeast is a carcinogen but it could be that some cancer-promoting agent may be produced in the fermenting process.

The present findings thus support the hypothesis that the consumption of beer made from maize is a factor in the development of oesophageal cancer in Africa. The weight of present evidence, however, suggests that any effect may occur through nutritional deficiencies that are engendered and through the promoting effects of alcohol rather than through the direct impact of a chemical carcinogen. It seems that the principal carcinogenic stimulus in the black South African populations that have been studied comes from tobacco and especially from pipe tobacco smoked either in pipes or in hand-rolled cigarettes.

This study was supported by a research grant from the National Cancer Association of South Africa.

The authors thank Dr M. Burr, MRC Epidemiology Unit (South Wales) and the late Dr Emanoel Lee, John Radcliffe Hospital, Oxford for their assistance.

### References

BARRELIER, M.T. (1974). *Le cancer de l'oesophage en Basse – Normandie*. These: Caen.

BRADSHAW, E. & SCHONLAND, M. (1969). Oesophageal cancer and lung cancer in Natal African males in relation to certain socio-economic factors. *Br. J. Cancer*, **23**, 275.

BRADSHAW, E. & SCHONLAND, M. (1974). Smoking, drinking and oesophageal cancer in African males of Johannesburg, South Africa. *Br. J. Cancer*, **30**, 157.

BRADSHAW, E., McGLASHAN, N.D., HARRINGTON, J.S. (1983). Oesophageal cancer: Smoking and drinking in the Transkei. Occasional paper, *27 Inst. Soc. & Econ. Res. Rhodes University*, Grahamstown, South Africa (*abstract*).

BRESLOW, N.E. & DAY, N.E. (1982). *Statistical methods in cancer research*, Vol. 1. *The analysis of case control studies*. IRAC: Lyon, p. 122.

COETZEE, A.M. (1978). Rook en gesondheid-feite en statistiek. *S. Afr. Med. J.*, **54**, 425.

COOK, P. (1971). Cancer of the oesophagus in Africa. *Br. J. Cancer*, **25**, 853.

COOK-MOZAFFARI, P. (1980). The epidemiology and pathology of cancer of the oesophagus. In *Recent Advances in Gastrointestinal Pathology*, Wright, R. (ed) p. 267. W.B. Saunders: London.

COOK-MOZAFFARI, P., AZORDEGAN, F., DAY, N.E., RESSICAUD, A., SABAI, C. & ARAMESH, B. (1979). Oesophageal cancer studies in the Caspian Littoral of Iran: Results of a case control study. *Br. J. Cancer*, **39**, 293.

DAY, N.E. & MUNOZ, N. (1982). Esophagus. In *Cancer epidemiology and prevention*, Schottenfeld, D. & Fraumeni, J.F. (eds) p. 596. W.B. Saunders: Philadelphia.

DAY, N.E. (1984). The geographic pathology of cancer of the oesophagus. *Br. Med. Bull.*, **40**, 329.

DOLL, R. (1971), Oesophageal cancer: A preventable disease? In *Int. Seminar on the epidemiology of oesophageal cancer*. Indian Cancer Society & UICC.

DOLL, R., MUIR, C. & WATERHOUSE, J. (eds) (1970). *Cancer Incidence in Five Continents*, Vol. II. Springer-Verlag: Berlin, p. 338.

HIGGINSON, J. & OETTLÉ, A.G. (1960). Cancer incidence in the Bantu and 'Cape Coloured' races of South Africa: Report of a cancer survey in the Transvaal (1953–55). *J. Natl Cancer Inst.*, **24**, 589.

HORMOZDIARI, H., DAY, N.E., ARAMESH, B. & MAHBOUBI, E. (1975). Dietary factors and oesophageal cancer in the Caspian Littoral of Iran, *Cancer Res.*, **35**, 3493.

HUNT, J.A. (1978). Squamous carcinoma of the oesophagus in urban South African blacks: A preliminary report of baseline studies. In *Carcinoma of the oesophagus*, Silber, W. (ed) p. 287. Balkema: Rotterdam.

KOLICHAVA, N.I. (1980). Epidemiology of oesophageal cancer in the USSR. In *Cancer epidemiology in the USA and USSR*, Levin, D.L. (ed). Washington Dept. Health and Human Services. NIH Publ. 80–2044, 191.

McGLASHAN, N.D., BRADSHAW, E. & HARRINGTON, J.S. (1982). Cancer of the oesophagus and the use of tobacco and alcoholic beverages in Transkei, 1975–1976. *Int. J. Cancer*, **29**, 249.

MORRIS, P. (1981). *Soweto, some factors, observations and guidelines for change*. Urban Foundation: Johannesburg.

MORRIS, P. & CELLIERS, S.P. (1980). *Soweto. Synopsis of a study by the Transvaal region of the Urban Foundation 1980*. Urban Foundation: Johannesburg.

NOVELLIE, L. (1968). Kaffir beer brewing. *Wallerstein Lab. Commun.*, **31**, 17.

NOVELLIE, L. (1986). Sorghum beer and related fermentations of Southern Africa. In *Indigenous fermented food of non-western origin*, Hesseltine, C.W. & Wang, H.L. (eds) *Mycologia Memoir* No. II, p. 219.

OETTLÉ, A.G. (1963). An epidemic of oesophageal carcinoma in Africa. *S. Afr. Med. J.*, **37**, 435.

OXFORD, T. (1926). The brewing of kaffir beer. *J. Inst. Brew.*, **32**, 314.

POTTERN, L.M., MORRIS, L.E., BLOT, W.J., ZIEGLER, R.G. & FRAUMENI, J.F. (1981). Oesophageal cancer among black men in Washington DC, I Alcohol, tobacco and other risk factors. *J. Natl Cancer Inst.*, **67**, 777.

ROSE, E.F. (1973). Oesophageal cancer in the Transkei 1935–69. *J. Natl Cancer Inst.*, **51**, 7.

ROSE, E.F. (1978). Patterns of occurrence of oesophageal cancer with particular reference to the Transkei. In *Carcinoma of the oesophagus*, Silber, W. (ed) p. 36. Balkema: Rotterdam.

SAS USERS GUIDE (1986), *Statistics*, Version 5. Edition, SAS Inc. p. 403.

SEGAL, I., LERIOS, M. & GRIEVE, T. (1984*a*). The emergence of chronic calcific pancreatitis in a developing country. In *Pancreatitis, Concepts and Classification*, Gyr, K. *et al.* (eds), p. 417. Excerpta Medica: Amsterdam.

SEGAL, I., WALKER, A.R.P., HAMILTON, D.G. & SOLOMON, A. (1984*b*). Cancer of the oesophagus in the South African black population. *Trop. Gastro.*, **5**, 20.

SOWETO: A SURVEY. Supplement. Financial Mail 25.3.83: 1.

SUGI SUPPLEMENTAL LIBRARY USERS GUIDE (1986). Version 5. Edition, SAS Inc., p. 269.

THURNHAM, D.I., ZHERG, S.F., MUNOZ, N. & 4 others (1985). Comparisons of riboflavin, vitamin A and zinc status of Chinese populations at high and low risk for oesophageal cancer. *Nutrition & Cancer*, **7**, 131.

TUYNS, A.J., PEQUINOT, G. & ABBATUCCI, J.S. (1977). Le cancer de l'oesophage en Ille-et-vilaine en fonction des niveaux de consommation d'alcool et de tabac. Des risques qui se multiplient. *Bull. Cancer*, **64**, 45.

VAN RENSBURG, S.J. (1981). Epidemiologic and dietary evidence for specific nutritional predisposition to oesophageal cancer. *J. Natl Cancer Inst.*, **67**, 243.

VAN RENSBURG, S.J., BENADE, A.S., ROSE, E.F. & DU PLESSIS, J.P. (1983). Nutritional status of African populations predisposed to oesophageal cancer. *Nutrition & Cancer*, **4**, 206.

VAN RENSBURG, S.J., BRADSHAW, E.S., BRADSHAW, D. & ROSE, E.F. (1985). Oesophageal cancer in Zulu men, South Africa: A case control study. *Br. J. Cancer*, **51**, 399.

WALKER, A.R.P. & ARVIDSSON, U.B. (1953). Iron overload in the South African Bantu. *Trans. R. Soc. Trop. Med. Hyg.*, **47**, 536.

WALKER, A.R.P., WALKER, B.F., ISAACSON, C., SEGAL, I. & PRYOR, S. (1984). Short duration of survival among South African Blacks with oesophageal cancer. *S. Afr. Med. J.*, **66**, 877.

ZAMMIT, I.V. (1980). The nutritive value of sorghum beer. *Sth. Afr. Food Review* (*Suppl*), **7**, 73.


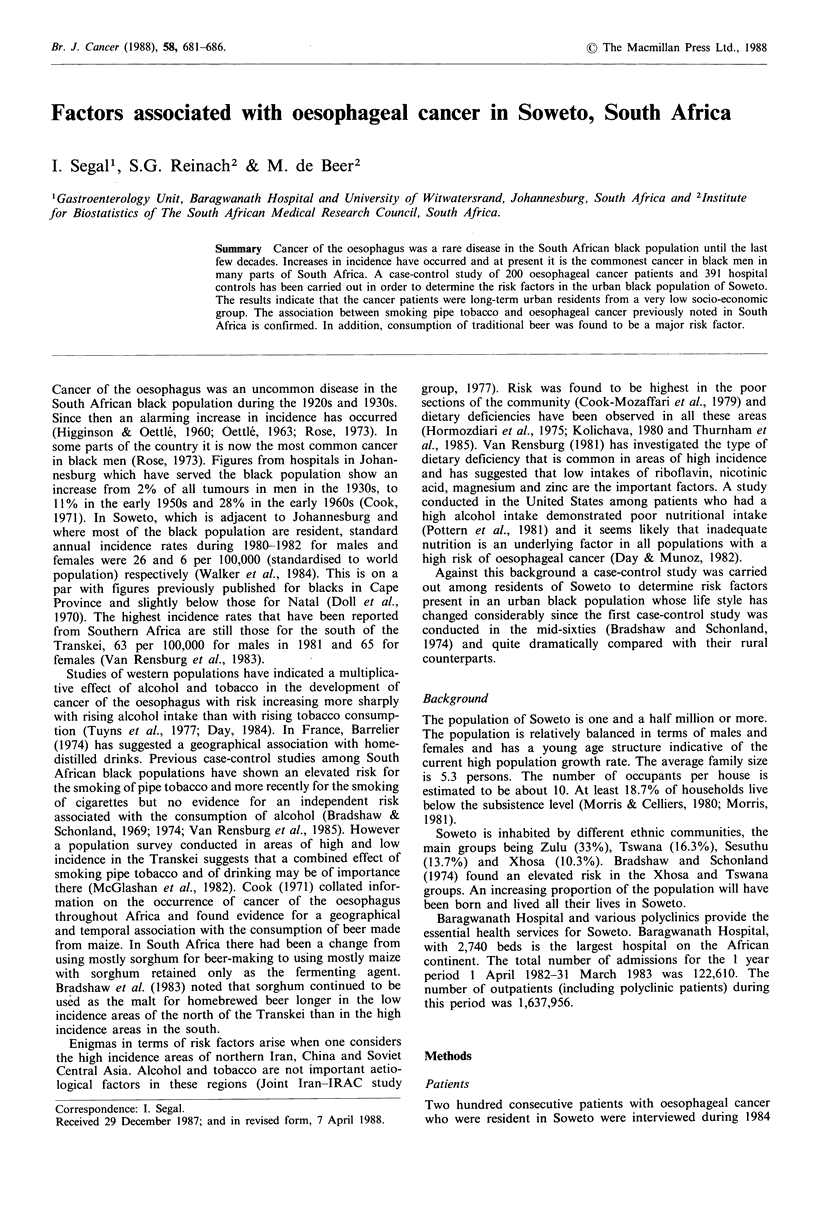

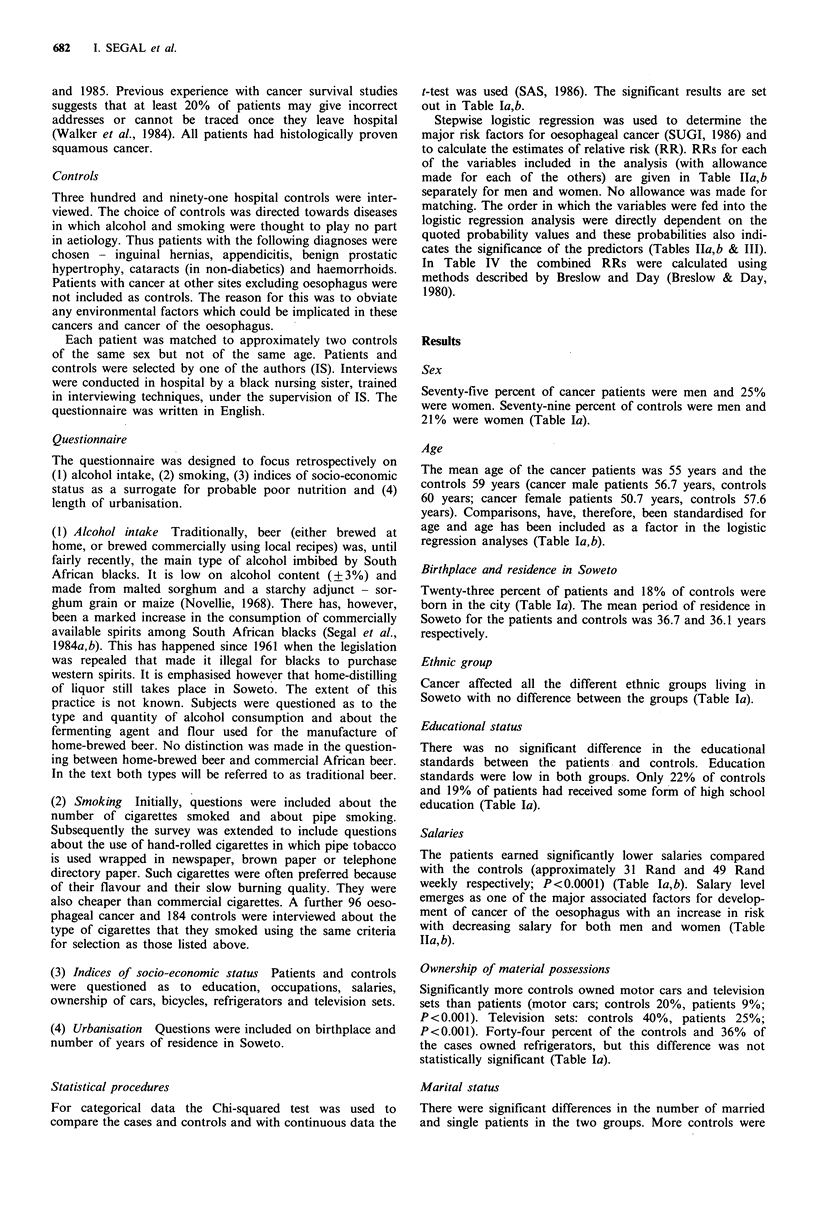

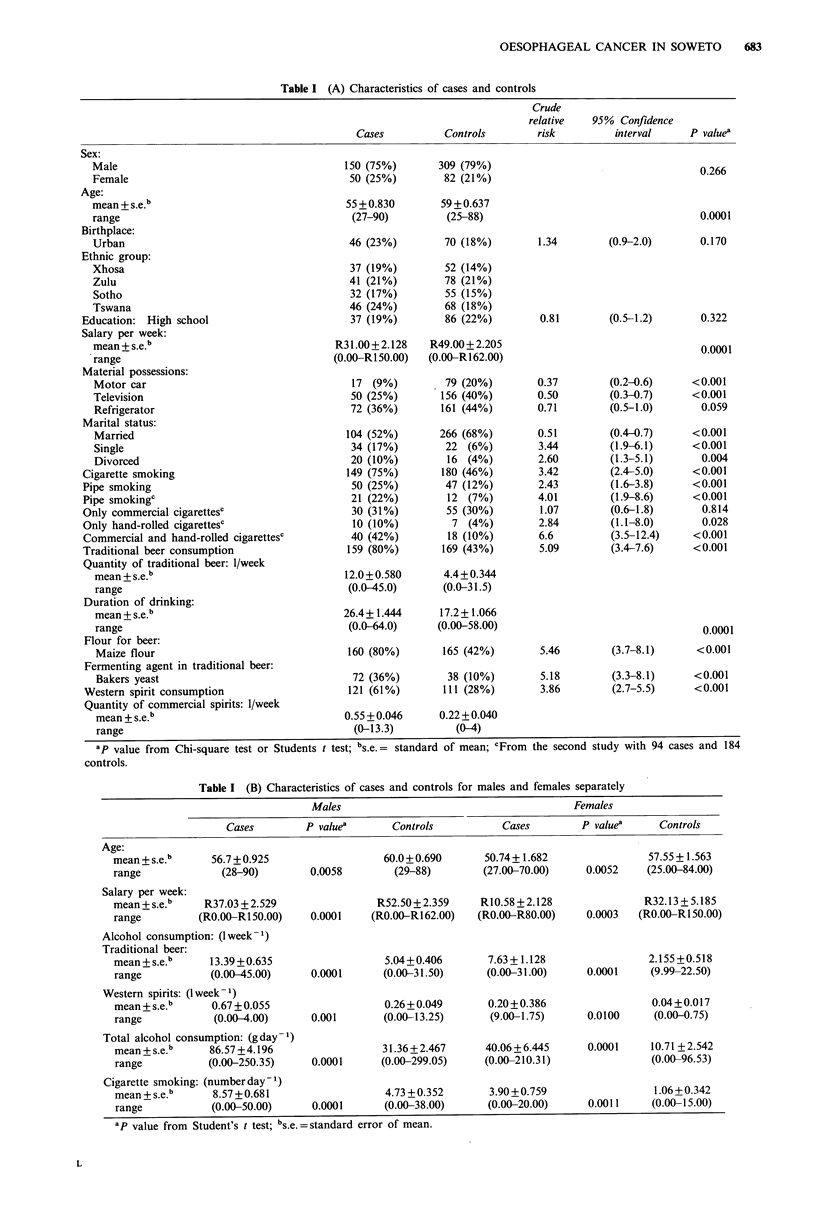

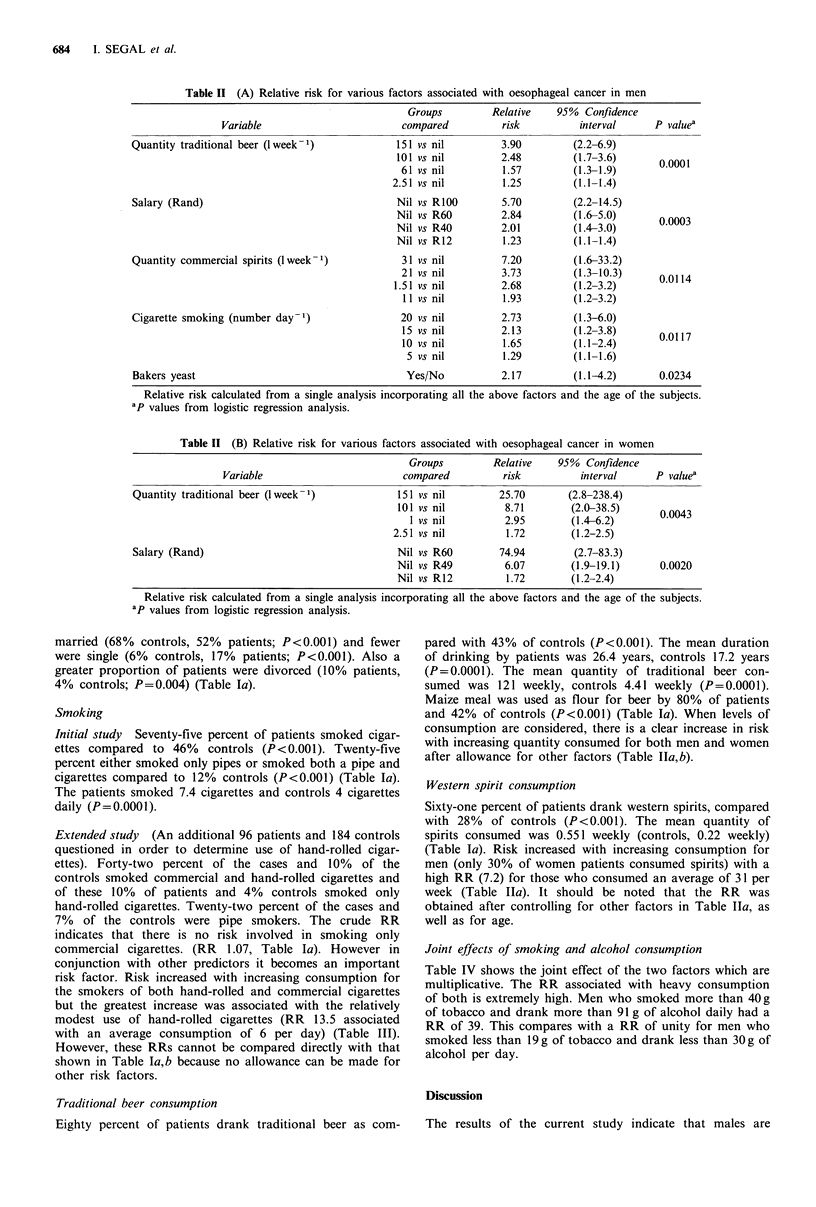

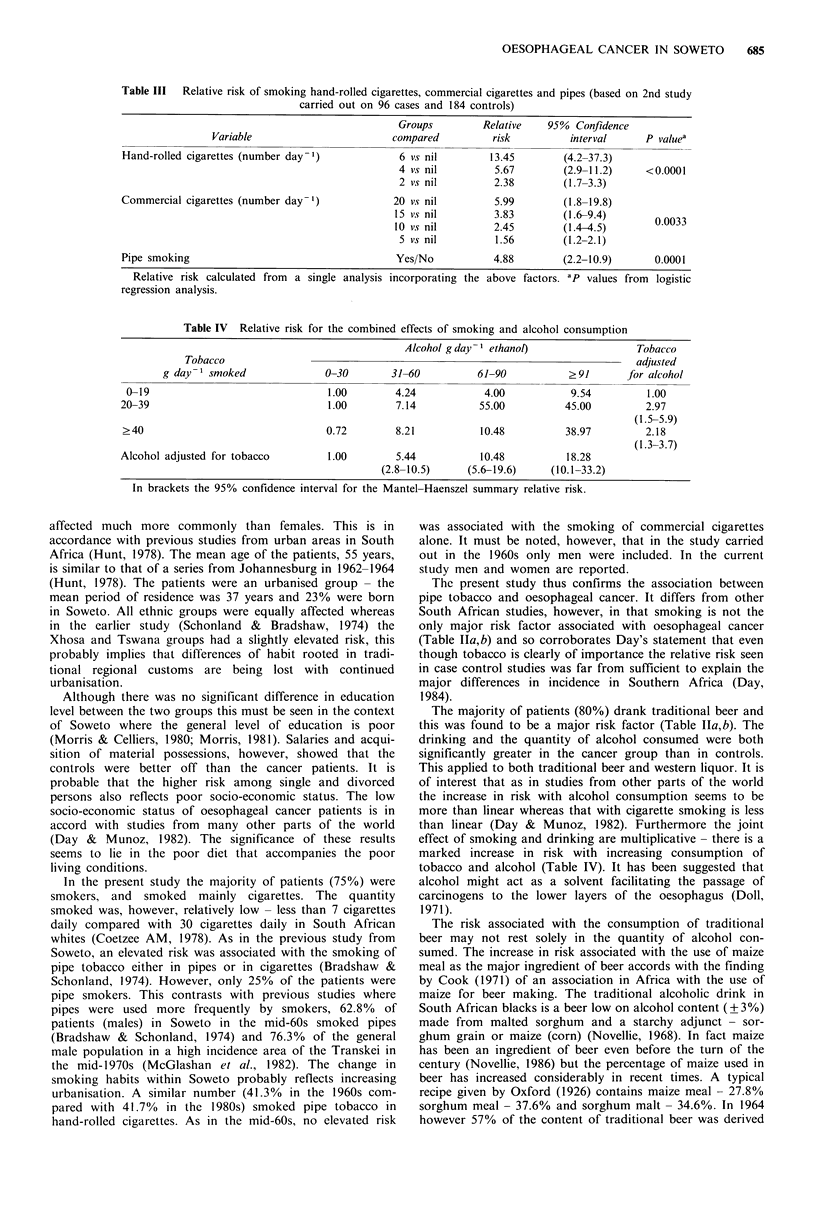

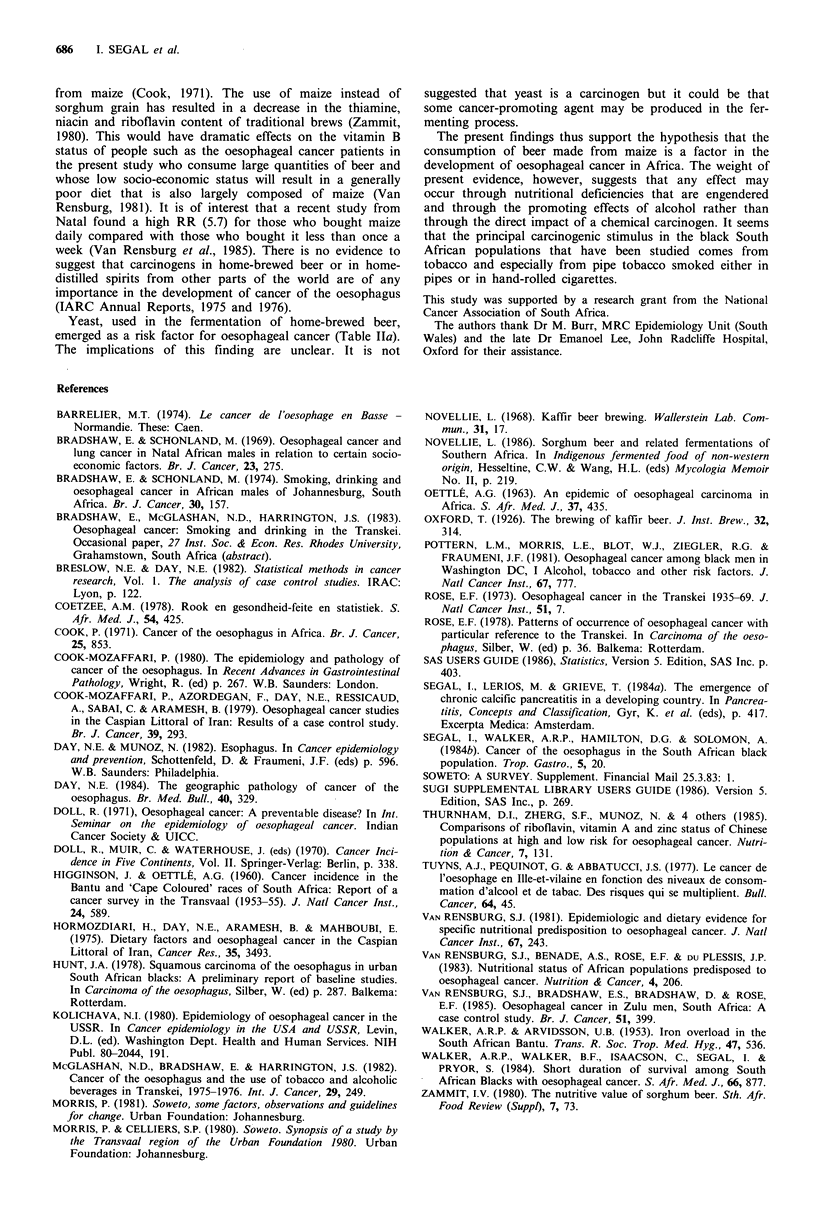


## References

[OCR_00943] Bradshaw E., Schonland M. (1969). Oesophageal and lung cancers in Natal African males in relation to certain socio-economic factors. An analysis of 484 interviews.. Br J Cancer.

[OCR_00948] Bradshaw E., Schonland M. (1974). Smoking, drinking and oesophageal cancer in African males of Johannesburg, South Africa.. Br J Cancer.

[OCR_00964] Coetzee A. M. (1978). Rook en gesondheid--feite en statistiek.. S Afr Med J.

[OCR_00977] Cook-Mozaffari P. J., Azordegan F., Day N. E., Ressicaud A., Sabai C., Aramesh B. (1979). Oesophageal cancer studies in the Caspian Littoral of Iran: results of a case-control study.. Br J Cancer.

[OCR_00968] Cook P. (1971). Cancer of the oesophagus in Africa. A summary and evaluation of the evidence for the frequency of occurrence, and a preliminary indication of the possible association with the consumption of alcoholic drinks made from maize.. Br J Cancer.

[OCR_00988] Day N. E. (1984). The geographic pathology of cancer of the oesophagus.. Br Med Bull.

[OCR_01000] HIGGINSON J., OETTLE A. G. (1960). Cancer incidence in the Bantu and "Cape Colored" races of South Africa: report of a cancer survey in the Transvaal (1953-55).. J Natl Cancer Inst.

[OCR_01006] Hormozdiari H., Day N. E., Aramesh B., Mahboubi E. (1975). Dietary factors and esophageal cancer in the Caspian Littoral of Iran.. Cancer Res.

[OCR_01023] McGlashan N. D., Bradshaw E., Harington J. S. (1982). Cancer of the oesophagus and the use of tobacco and alcoholic beverages in Transkei, 1975-6.. Int J Cancer.

[OCR_01055] Pottern L. M., Morris L. E., Blot W. J., Ziegler R. G., Fraumeni J. F. (1981). Esophageal cancer among black men in Washington, D.C. I. Alcohol, tobacco, and other risk factors.. J Natl Cancer Inst.

[OCR_01061] Rose E. F. (1973). Esophageal cancer in the Transkei: 1955-69.. J Natl Cancer Inst.

[OCR_01080] Segal I., Walker A. R., Hamilton D. G., Solomon A. (1984). Cancer of the oesophagus in the South African black population.. Trop Gastroenterol.

[OCR_01091] Thurnham D. I., Zheng S. F., Munoz N., Crespi M., Grassi A., Hambidge K. M., Chai T. F. (1985). Comparison of riboflavin, vitamin A, and zinc status of Chinese populations at high and low risk for esophageal cancer.. Nutr Cancer.

[OCR_01097] Tuyns A. J., Péquignot G., Jensen O. M. (1977). Le cancer de l'oesophage en Ille-et-Vilaine en fonction des niveaux de consommation d'alcool et de tabac. Des risques qui se multiplient. Bull Cancer.

[OCR_01113] Van Rensburg S. J., Bradshaw E. S., Bradshaw D., Rose E. F. (1985). Oesophageal cancer in Zulu men, South Africa: a case-control study.. Br J Cancer.

[OCR_01118] WALKER A. R., ARVIDSSON U. B. (1953). Iron overload in the South African Bantu.. Trans R Soc Trop Med Hyg.

[OCR_01121] Walker A. R., Walker B. F., Isaacson C., Segal I., Pryor S. (1984). Short duration of survival among South African blacks with oesophageal cancer.. S Afr Med J.

[OCR_01108] van Rensburg S. J., Benadé A. S., Rose E. F., du Plessis J. P. (1983). Nutritional status of African populations predisposed to esophageal cancer.. Nutr Cancer.

[OCR_01103] van Rensburg S. J. (1981). Epidemiologic and dietary evidence for a specific nutritional predisposition to esophageal cancer.. J Natl Cancer Inst.

